# Different effects of progesterone and estradiol on chimeric and wild type aldosterone synthase *in vitro*

**DOI:** 10.1186/1477-7827-11-76

**Published:** 2013-08-13

**Authors:** Andrea Vecchiola, Carlos F Lagos, Cristóbal A Fuentes, Fidel Allende, Carmen Campino, Carolina Valdivia, Alejandra Tapia-Castillo, Tadashi Ogishima, Kuniaki Mukai, Gareth Owen, Sandra Solari, Cristian A Carvajal, Carlos E Fardella

**Affiliations:** 1 Molecular Endocrinology Laboratory, Department of Endocrinology, School of Medicine, Pontificia Universidad Catolica de Chile, Lira 85, 5th Floor, Santiago, Chile; 2 Department of Pharmacy, Faculty of Chemistry, Pontificia Universidad Catolica de Chile, Av. Vicuña Mackenna 4860, Macul, Santiago, Chile; 3 Department of Clinical Laboratories, School of Medicine, Pontificia Universidad Catolica de Chile, Av. Vicuña Mackenna 4860, Macul, Santiago, Chile; 4 Department of Chemistry, Faculty of Sciences, Kyushu University, 6-10-1 Hakozaki, Higashi-ku, Fukuoka, 812-8581, Japan; 5 Department of Biochemistry, School of Medicine, Keio University, 35 Shinanomachi, Shinjuku-ku, Tokyo, 160-8582, Japan; 6 Department of Physiology, Faculty of Biological Sciences, Pontificia Universidad Catolica de Chile, Portugal 45, Santiago, Chile; 7 Millennium Institute of Immunology and Immunotherapy, Santiago, Chile

**Keywords:** Familial hyperaldosteronism type I, Aldosterone synthase, Chimeric CYP11B1/B2 gene, *In vitro* assay, Molecular modelling

## Abstract

**Background:**

Familial hyperaldosteronism type I (FH-I) is caused by the unequal recombination between the 11beta-hydroxylase (CYP11B1) and aldosterone synthase (CYP11B2) genes, resulting in the generation of a CYP11B1/B2 chimeric gene and abnormal adrenal aldosterone production. Affected patients usually show severe hypertension and an elevated frequency of stroke at a young age. Aldosterone levels rise during pregnancy, yet in pregnant women with FH-1, their hypertensive condition either remains unchanged or may even improve. The purpose of this study was to investigate *in vitro* whether female sex steroids modulate the activity of chimeric (ASCE) or wild type (ASWT) aldosterone synthase enzymes.

**Methods:**

We designed an *in vitro* assay using HEK-293 cell line transiently transfected with vectors containing the full ASCE or ASWT cDNAs. Progesterone or estradiol effects on AS enzyme activities were evaluated in transfected cells incubated with deoxycorticosterone (DOC) alone or DOC plus increasing doses of these steroids.

**Results:**

In our *in vitro* model, both enzymes showed similar apparent kinetic parameters (Km = 1.191 microM and Vmax = 27.08 microM/24 h for ASCE and Km = 1.163 microM and Vmax = 36.98 microM/24 h for ASWT; p = ns, Mann–Whitney test). Progesterone inhibited aldosterone production by ASCE- and ASWT-transfected cells, while estradiol demonstrated no effect. Progesterone acted as a competitive inhibitor for both enzymes. Molecular modelling studies and binding affinity estimations indicate that progesterone might bind to the substrate site in both ASCE and ASWT, supporting the idea that this steroid could regulate these enzymatic activities and contribute to the decay of aldosterone synthase activity in chimeric gene-positive patients.

**Conclusions:**

Our results show an inhibitory action of progesterone in the aldosterone synthesis by chimeric or wild type aldosterone synthase enzymes. This is a novel regulatory mechanism of progesterone action, which could be involved in protecting pregnant women with FH-1 against hypertension. *In vitro*, both enzymes showed comparable kinetic parameters, but ASWT was more strongly inhibited than ASCE. This study implicates a new role for progesterone in the regulation of aldosterone levels that could contribute, along with other factors, to the maintenance of an adequate aldosterone-progesterone balance in pregnancy.

## Background

Primary aldosteronism is the most common form of secondary hypertension, with an estimated prevalence of 10% in referred patients and 4% in primary care [1,2] but as high as 20% in patients with resistant hypertension [3,4]. Primary aldosteronism is characterised by hypertension with low plasma renin activity and elevated aldosterone levels that are often observed with hypokalemia and abnormal adrenal steroid production [5].

Familial hyperaldosteronism type I (FH-I) occurs by an unequal crossing-over of the genes encoding steroid 11β-hydroxylase (CYP11B1) and aldosterone synthase (CYP11B2), resulting in a chimeric CYP11B1/B2 gene that produces an enzyme with aldosterone synthase activity with ectopic expression in the zona fasciculata, which is regulated by plasma adrenocorticotrophic hormone (ACTH) levels instead of by angiotensin II [6-8]. As a consequence, aldosterone, 18-hydroxycortisol (18OHF), and 18-oxocortisol (18oxoF) are produced. Different FH-I pedigrees exhibit different crossover points between intron 2 and exon 4, suggesting that the mutations arise independently in each pedigree [9-11]. Exons 5 and 6 of CYP11B2 are required for aldosterone, 18OHF, and 18oxoF production [12,13].

There is limited information about pregnancy in FH-1 women. It is a known fact that normal pregnancy is characterised by an increase in maternal plasma volume which is mediated, at least in part, by the activation of the maternal renin-angiotensin system with increased levels of renin activity, angiotensin II and aldosterone. Furthermore, Gennari-Moser et al. recently demonstrated that vascular endothelial growth factor (VEGF) stimulates aldosterone synthesis in H295R adrenal cells as assessed by the conversion of 3H-deoxycorticosterone (DOC) to 3H-aldosterone. This novel mechanism may also be operating during gestation [14]. During the first trimester of pregnancy, aldosterone has a proliferative effect on trophoblast in addition to causing a volume expansion to allow the foetus to develop [15]. On the other hand, progesterone has pleiotropic actions; for instance, it can increase the synthesis of aldosterone because is a substrate for 21-hydroxylase [16] and also increase the mRNA levels of CYP11B2 in rats [17]. Progesterone also has an antagonist effect because it competes with aldosterone by binding to the mineralocorticoid receptor (MR) [18]. Some authors have speculated that MR activation by DOC may be prevented by a pre-receptor protective mechanism under normal circumstances, although its nature is unclear [14,19]. Our findings suggest that there may be a close relationship between the levels of these steroids, which must be carefully regulated throughout pregnancy to prevent hypertension, and reaching a successful delivery and a healthy newborn.

Because aldosterone, progesterone and estradiol increased several fold during gestation but FH-1 pregnant women did not experience a worsened hypertensive condition, we hypothesised that sexual steroids might modulate the activity of the chimeric and wild type aldosterone synthase enzymes. Accordingly, we investigated this hypothesis using an *in vitro* system.

The aims of this study were: a) to carry out an *in vitro* assay to evaluate the activity of these enzymes using HEK-293 cells line transfected with chimeric and wild type aldosterone synthase enzymes, b) to investigate whether progesterone and estradiol inhibits chimeric and wild type aldosterone synthase enzymes in our *in vitro* assay, and c) to examine the putative binding mode of these steroids to chimeric and wild type aldosterone synthase enzymes by molecular modelling studies.

## Methods

### Synthesis of the chimeric CYP11B1/B2 gene

Recently, we reported the unequal crossover break point in the CYP11B1/B2 gene [20]. The 50-bp crossover region contains segments of intron 3 of CYP11B1 (c.2937-40) and exon 4 of CYP11B2 (c.2937 + 10). Using the PCMV-CYP11B1 and PCMV-CYP11B2 vectors and based on restriction enzyme analysis, we selected a crossover point to create the fusion vector containing exons 1 to 3 of CYP11B1 (1-573 bp) and exon 4 to 9 of CYP11B2 (574-1512 bp). Exons 5 and 6 of CYP11B2 were maintained in the chimeric enzyme. PCMV-CYP11B1 and PCMV-CYP11B2 vectors were kindly provided by Dr. Walter L. Miller (University of California, San Francisco). Mutagenex Inc. (Hillsborough, NJ, USA) [21] performed the chimeragenesis of the CYP11B1/B2 gene. Restriction endonuclease digestion and Sanger sequencing confirmed the integrity of the plasmid constructs, and the amplification products were verified by sequencing at Macrogen (Rockvill, MD, USA) [22].

### Cell culture and transient transfections

The human embryonic kidney cell line, HEK-293, was grown in high-glucose Dulbecco’s modified Eagle’s medium (DMEM HG, Life Technologies, Sao Paulo, Brazil) supplemented with 10% foetal bovine serum (FBS, Life Technologies, Sao Paulo, Brazil), 100 IU/mL penicillin and 100 μg/mL streptomycin. For the enzyme activity studies, DMEM HG-FBS medium was treated with activated charcoal to eliminate steroid contaminants. For the transfection experiments, HEK-293 cells were plated at 6×10^5^ cells per well in 6-well plates and then transfected using Turbofect™ *in vitro* transfection reagent (Fermentas, Thermo Scientific, Rockford, IL, USA), in accordance with the manufacturer's protocol. Briefly, 2 μg of pCMV4, pCMV4-CYP11B1, pCMV4-CYP11B2, or pCMV4-CYP11B1/B2 plasmid were added in DMEM. pZsGreen1-n1 (0.3 μg, Clontech, California, USA) was added as a marker of transfection efficiency. The transfected cells were visualised using a Panasonic–DMC-LC40 LUMIX and the Pro-QV7software. The efficiency of transfection was evaluated using an Olympus CKX41 inverted microscope coupled to an Olympus U-RFL-T. For this purpose, we took six photographs (40×) of plates containing HEK-293 cells (in each condition) under bright field and again with the same field of view under fluorescent light using a Micropublisher 3.3RTV camera and the Pro-QV7software. Then, the total number of cells was counted under both conditions using the Image J v1.46 program [18] in three independent trials.

### Sodium dodecylsulfate–polyacrylamide gel electrophoresis (SDS-PAGE) and Western blotting

The whole-cell extracts of untransfected HEK-293, PCMV-CYP11B1, PCMV-CYP11B1/B2 or PCMV-CYP11B2-transfected cells were obtained using 100 μL of lysis buffer containing protease inhibitors. The protein concentration was estimated using the BCA protein colorimetric assay kit (Thermo Scientific, Rockford, IL, USA) on an Infinite® 200 PRO NanoQuantmultimode reader (Tecan, Männedorf, Switzerland). The denatured protein samples (50 μg per well) were separated by 12% SDS-PAGE and were immobilised onto 0.45-μm-pore nitrocellulose membranes (Thermo Scientific, Rockford, IL, USA). The membranes were probed with a rabbit anti-human CYP11B2 antibody (aa 80–90 (RYNLGGPRMVC of CYP11B2) followed by a goat anti-rabbit peroxidase-conjugated antibody (Thermo Scientific, Rockford, IL, USA), as previously described [23]. The CYP11B2 protein was visualised using the enhanced chemiluminescence Super Signal Pico Chemiluminescent Substrate kit (Thermo Scientific, Rockford, IL, USA) and Agfa X-ray film (Agfa-Gevaert, N.V, Mortsel, Belgium). Protein extracts from a sample of human adrenal gland tissue were used as positive control. The loading control was β-actin. The CYP11B1/B2 has an amino acid sequence of 1–191 from the N-terminus of the CYP11B1 enzyme and was thus not detected by this antibody.

### Expression of CYP11B1/B2 and CYP11B2

Expression of CYP11B2 and CYP11B1/B2 in transfected HEK-293 cells were evaluated by qRT-PCR. Total RNA was extracted from transfected HEK-293 cells treated or not with progesterone by TRIZOL® (Life Technologies, California, USA) then reverse transcribed using RevertAid H Minus Reverse Transcriptase (Thermo Scientific, California, USA) following the manufacture’s instruction. Quantitative real-time polymerase chain reaction was performed using Maxima SYBR (Thermo Scientific, California, USA). Primers were CYP11B2 forward: 5`- gga act tcc acc acg tgc cct tt-3` and CYP11B2 reverse: 5`- att gag gcc tgg cac gtc cc-3. GAPDH forward: 5-gaa cat cat ccc tgc ctc tac t −3`, and GAPDH reverse: 5 –cct gct tca cc acct tct tg −3. The mRNA expression was quantified by ΔΔCt method relative to that of GAPDH [24]. CYP11B2 primers are located in exons eight and nine.

### Aldosterone synthase activity assay

To determine the kinetic constants under our assay conditions, the PCMV-CYP11B1, PCMV-CYP11B1/B2, and PCMV-CYP11B2 HEK-293 transfected cells at 18 h post-transfection were incubated for 24 h with increasing concentrations (ranging from 0.18 to 30 μmol/L) of deoxycorticosterone as substrate (DOC, Steraloids Inc., Andover, MA, USA). Resultant aldosterone production was quantified using HPLC-MS/MS (Agilent 1200, ABI Sciex API4000 Qtrap). The apparent kinetics parameters Km and Vmax were determined by plotting the aldosterone production versus the corresponding substrate concentrations and applying Michaelis–Menten kinetics using the Prism v5.03 program (GraphPad Software, Inc.).

### Aldosterone synthase inhibitory assay

All chemicals were purchased from Sigma (Sigma-Aldrich Quimica Ltda, Santiago, Chile). To determine any inhibition by sex steroid hormones, at 18 h post-transfection the cells were washed with PBS and 1.5 μmol/L DOC-DMEM steroid hormone-depleted FBS and exposed to 24 hours of increasing concentrations of progesterone or estradiol (0.625-10.0 μM). Ketoconazole (ranging from 0.625 to 5 μM) was used as an inhibitor control. The supernatant (1.0 mL) was collected, and aldosterone levels were measured by HPLC-MS/MS in four independent trials. The IC50 was determined by plotting the aldosterone production versus the corresponding log inhibitor concentrations and applying a dose–response inhibition analysis.

### Effect of progesterone on chimeric and wild type aldosterone synthase activity

To determine the effect of progesterone on apparent kinetic constants under our assay conditions, the PCMV-CYP11B1/B2, and PCMV-CYP11B2 HEK-293 transfected cells, after 18 h post-transfection, were incubated for 24 h with increasing concentrations (ranging from 0.18 to 30 μM) of DOC (Steraloids Inc., Andover, MA, USA) and progesterone at the IC50 concentrations for each enzyme. The resultant aldosterone production in the supernatant was quantified using HPLC-MS/MS (Agilent 1200, ABI Sciex API4000 Qtrap). The apparent kinetics parameters Km and Vmax were determined by plotting the aldosterone production versus the corresponding substrate concentrations and applying Michaelis–Menten kinetics using the Prism v5.03 program (GraphPad Software, Inc.).

### Cell viability assay

The CellTiter 96 AQueous One Solution Cell Proliferation Assay (Promega) kit containing the tetrazolium compound MTS ([3-(4,5-dimethyl-2-yl)-5-(3-carboxymethoxyphenyl)-2-(4-sulfophenyl)-2H-tetrazolium, inner salt]) was used to monitor cell viability according to the manufacturer’s protocols. Briefly, human embryonic kidney (HEK-293) cells maintained in DMEM with 10% foetal bovine serum (FBS) under standard cell culture conditions (37°C, humidified, 5% CO2) were plated at a density of 1.5×10^4^ cells/well in a 96-well plate and incubated in growth media for 18 hours. Cells were treated with 0.8 to-50 μM of progesterone, estradiol or DOC in DMEM containing 10% FBS for 24 hours. After the indicated time of incubation with the appropriate medium, a 20 μL MTS/PMS (1:0.05) mixture was added per well, and cells were incubated for an additional hour. MTS is reduced by viable cells to formazan, which was monitored at 490 nm by an ELX-800 universal plate reader (BioTek, Winooski, VT). Formazan production is time dependent and proportional to the number of viable cells. Cells not incubated in DMEM were used as control condition. The percentage of cell death was calculated by the ratio of the optical density obtained in each treatment to that obtained for the controls. A cytotoxic concentration was noted when the optical density in each condition was less than the average of the control minus two standard deviations.

### Molecular modelling of CYP11B1 and CYP11B1/B2 chimeric proteins and steroids docking

The amino acid sequence of the human CYP11B1 was retrieved from the Uniprot database (entry code P15538), and the chimeric CYP11B1/B2 sequence was obtained by performing DNA sequencing [25]. The comparative modelling was performed using the MODELLER program implemented in the Build Homology Models protocol in Discovery Studio v2.1 (Accelrys Inc., San Diego, USA) [26] using the recently reported crystal structure of human CYP11B2 in complex with DOC, which was identified as a suitable template for the modelling of both proteins (PDB id 4DVQ, resolution 2.49 Å) [27]. For modelling purposes, the first 33 residues of each protein were not included. The coordinates for DOC and the HEME group were modelled using the copy ligand parameter from the template structure, from which the Chain A was used. One hundred models for each protein were generated, and the top ranked by the MODELLER internal DOPE score energy minimized using the conjugate gradient algorithm until a RMS gradient of 0.001 kcal/mol Å was reached. The CHARMM22 force field with a dielectric constant of 4 and a distance-dependent dielectric implicit solvent model was used to mimic the membrane environment [28]. Model quality was assessed by Ramachandran plot analysis, PROSA and Verify3D structure validation [29-31]. The electrostatic potential energy profiles were calculated using APBS [32]. Conformers of each docked compound were obtained with OMEGA v2.4.6 using default parameters [33,34]. Docking calculations were performed using FRED v3.0 (OpenEye Scientific Software, Santa Fe, New Mexico) [35], and the solutions ranked according to the Chemgauss4 scoring function [36].

### Data analysis

Data are expressed as the mean +/- SEM. The kinetic parameters Vmax and Km were obtained by Prism v5.03. Differences between the means were analysed by repeated measures of an ANOVA and Tukey’s post hoc test. Differences of area under curve analyses were performed by the Mann Whitney test. Statistical analysis was performed using Prism v5.03 (GraphPad Software, Inc.). Differences were considered significant at p < 0.05.

## Results

### Design of vectors and chimeragenesis

**Figure 1:**
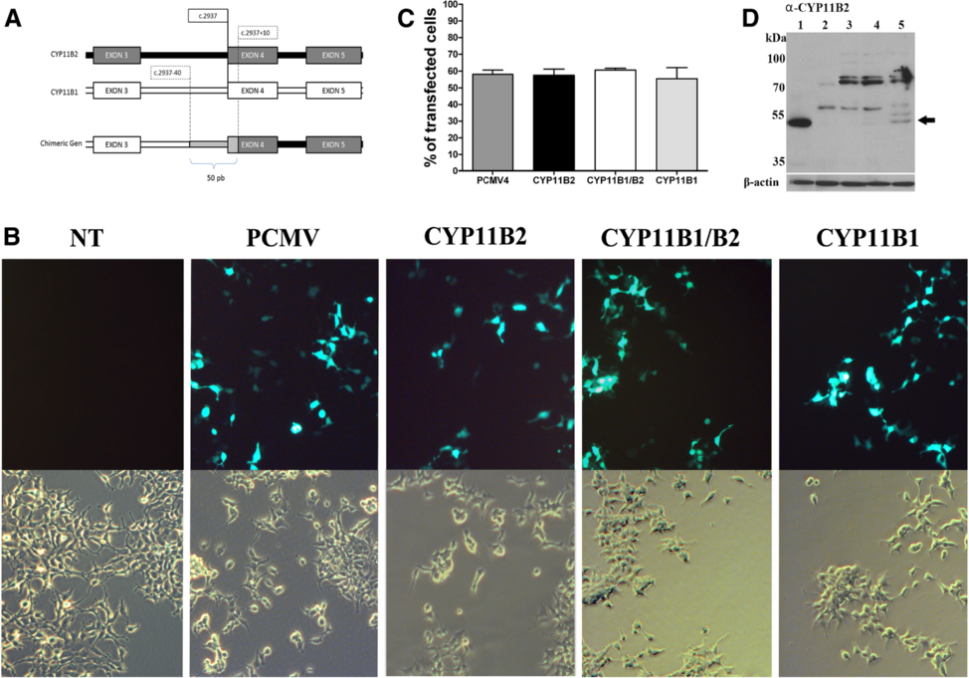
**Expression of aldosterone production by ASCE and ASWT *in vitro. *A)** Schematic representation of the CYP11B1/B2 chimeric gene, showing the crossover region between intron 3 of CYP11B1 and exon 4 of CYP11B2. **B)** A representative image of the transfection efficiency. Green fluorescence (upper panel) and the same bright field (lower panel) of transfected or non-transfected HEK-293 (NT). **C)** An average transfection efficiency of 50 percent was observed from three independent experiments for each construct. **D)** A representative Western blot of CYP11B2. Line 1, human adrenocortical adenoma; line 2, NT-HEK-293; line3, PCMV-CYP11B1 transfected HEK-293; line 4, PCMV-CYP11B1/B2; and line 5, PCMV-CYP11B2 transfected HEK-293. Actin was used as a loading control.

As described in the methods section, the crossover point was used in the chimeragenesis to create the fusion vector CYP11B1 (1-573 bp)/CYP11B2 (574-1512 bp). A schematic representation of the FH-I crossover and the resultant ASCE product that was synthesised for assay studies is shown in Figure 1A.

### *In vitro* expressed ASCE displayed similar aldosterone production to ASWT

A representative image of HEK-293 transfected with wild type or chimeric expression vectors are shown in Figure 1B. Morphological changes were not observed between the HEK-293 cells transfected with the constructs containing the CYP11B enzymes and the cells transfected with the PCMV vector or the non-transfected cells (NT). Comparable transfection efficiencies were observed by counting cells that express the green fluorescent protein, which correlated with the total number of cells for each assay condition (Figure 1C). The expression of the aldosterone synthase in HEK-293 cells transfected with the constructs containing the CYP11B2 was examined by Western blotting (Figure 1D). A human adenoma sample from the adrenal cortex was used as positive control (line 1), and the following were observed: no transfected HEK-293 (line 2); PCMV-CYP11B1 (line 3); PCMV-CYP11B1/B2 (line 4) and PCMV-CYP11B2 (line 5). An intense immunoreactive band with an apparent molecular weight of approximately 50 kDa was present in the human adenoma sample and in the HEK-293 cells transfected with PCMV-CYP11B2. Western blotting using a CYP11B1 antibody was performed for the same samples. Unfortunately, the immunoreactivity band was too weak. To probe the CYP11B1 activity of PCMV-CYP11B1 construction, we incubated PCMV-CYP11B1 transfected HEK-293 cells with increasing concentrations of corticosterone (0.6-1.5 μM), and we obtained the expected increasing levels of cortisol (data not shown).

### ASCE and ASWT displayed similar calculated kinetics enzymatic parameters *in vitro*

**Figure 2:**
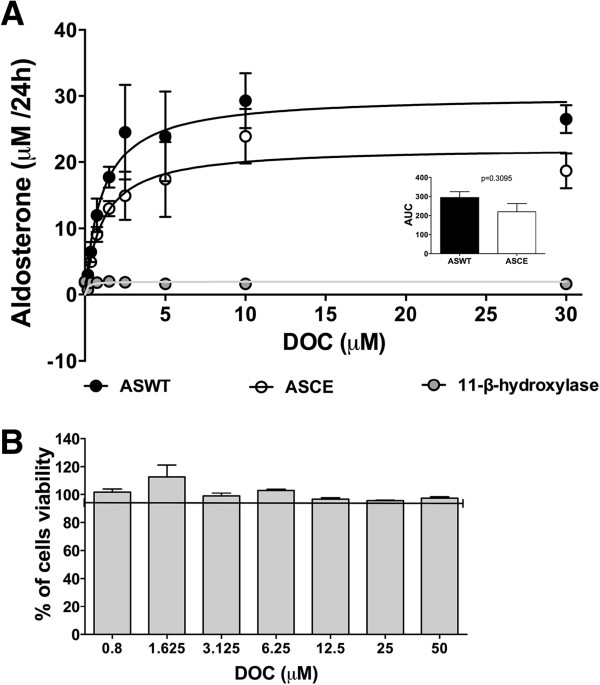
**Enzyme kinetics of aldosterone production by ASCE and ASWT *in vitro. *A)** Dose response curves of aldosterone production catalysed by ASCE (open circles), ASWT (closed circles) and 11β-hydroxylase (grey circles) incubated with 11-deoxycorticosterone (DOC 0.18-30 μM) for 24 h. Data are expressed as the mean +/− S.E.M. of 5 independent trials. The apparent kinetic enzyme parameters obtained were Km = 1.191 μM and Vmax = 27.08 μM/24 h for ASCE and Km = 1.163 μM and Vmax = 36.98 μM/24 h for ASWT, and the area under curve for each of the enzyme activities (ANOVA and Mann–Whitney test, p = 0.3095) is shown within the graph. **B)** Cell viability of HEK-293 incubated with increasing doses of DOC (0.8-50 μM).

The transfected HEK-293 cells supported CYP11B2- and CYP11B1/B2-dependent steroid conversion without the additional heterologous expression of the corresponding electron donor system, similar to previous experiments reported by Denner et al. [37]. Figure 2A shows a dose response curve performed for five independent HEK-293 transfections of PCMV-CYP11B1/B2, PCMV-CYP11B2 or PCMV-CYP11B1 (11β-hydroxylase gene, 11BH), incubated with increasing concentrations of DOC (0.18-30 μM) for 24 hours. The aldosterone production versus substrate concentration was plotted for ASCE (open circles), ASWT (closed circles) and 11BH (grey circles). 11β-hydroxylase did not produce aldosterone in any of the DOC concentrations that were probed. The apparent kinetic enzyme parameters obtained were Km = 1.191 μM and Vmax = 27.08 μM/24 h for ASCE and Km = 1.163 μM and Vmax = 36.98. The comparison of the area under the curve for aldosterone production by ASCE and ASWT did not show any significant differences (p = 0.3095 Mann–Whitney test). This finding suggests that both enzymes exhibited analogous affinity for the substrate and showed the same efficiency to produce aldosterone. DOC did not exhibit a toxic effect at any concentration probed (Figure 2B). Non-transfected HEK-293 cells incubated with DOC (1.5 μM) did not produce aldosterone (data not shown).

### Aldosterone production by ASCE and ASWT was inhibited by progesterone but not by estradiol

**Figure 3:**
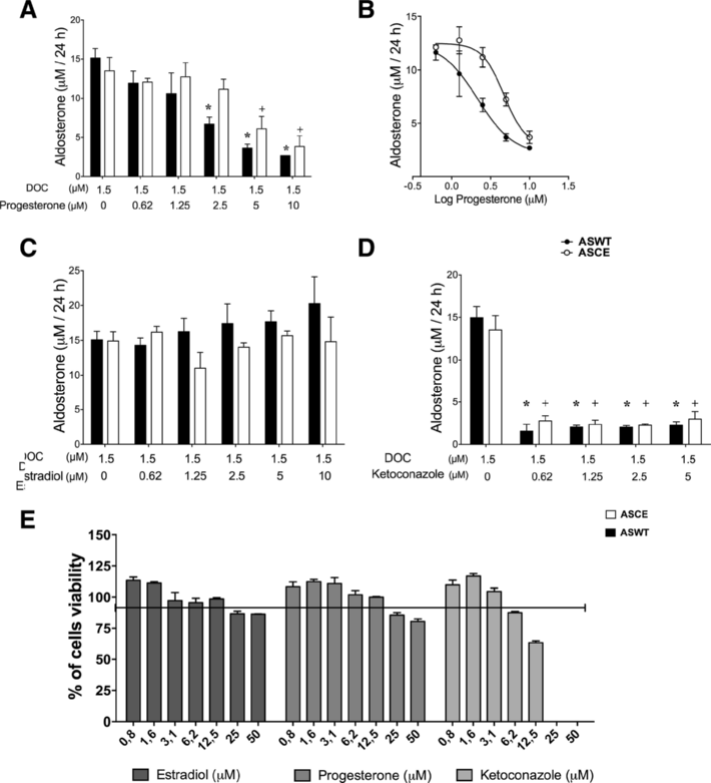
**Dose response effect of steroids on DOC-incubated release of aldosterone by PCMV-CYP11B2- or chimeric PCMV-CYP11B1/B2-transfected HEK-293 cells.** The mean aldosterone production by ASCE was 13.6 μM/24 h and by ASWT was 15 μM/24 h, when incubated with 1.5 μM of DOC. **A)** Inhibition of ASCE by progesterone was statistically significant from 5 μM and for ASWT from 2.5 μM (+ and *, p > 0.01). **B)** Dose response curve for aldosterone production by ASCE and ASWT (μM/24 h) versus the logarithm of the progesterone concentration. A calculated IC50 value of 3.907 μM for ASCE and 2.240 μM for ASWT was obtained. **C)** Estradiol shows no inhibitory activity on the ASCE or ASWT enzymes in the range of concentrations probed. **D)** Ketoconazole inhibited both enzymes by 90% at all concentrations probed (p < 0.001). **E)** Cell viability of HEK-293 incubated with increasing doses of progesterone and estradiol (progesterone and estradiol or ketoconazole) (0.8-50 μM). Data are expressed as the mean +/− S.E.M. of 4 independent trials.

The average aldosterone production by ASCE was 13.6 μM/24 h and by ASWT was 15 μM/24 h when the enzymes were incubated with 1.5 μM of DOC. In the presence of DOC as substrate, progesterone inhibited the aldosterone production by both ASCE and ASWT in a dose-dependent manner with similar efficacies. Statistically significant inhibition of ASCE was achieved at 5 μM of progesterone (Figure 3A, white bars), and statistically significant inhibition of ASWT was achieved at 2.5 μM of progesterone (Figure 3A, black bars). For ASCE, the calculated IC50 value for progesterone was 3.907 μM, and for ASWT, it was 2.240 μM (Figure 3B). These inhibition values were not due to an inhibition in plasmid transcription efficiency (See Additional file 1: Figure S1). Estradiol did not affect ASCE or ASWT aldosterone synthase activity in the range of concentrations assayed (Figure 3C) or coincubated with progesterone (See Additional file 2: Figure S2). As expected, the known aldosterone synthase inhibitor ketoconazole inhibited both enzyme activities by 90% at all concentrations probed (Figure 3D). None of the steroids assayed nor ketoconazole demonstrated cytotoxic effects in HEK-293 cells in any of the concentrations that were probed (Figure 3E). As a control, non-transfected HEK-293 cells were treated with the same increasing concentrations of progesterone or estradiol. No aldosterone production was detected under these conditions (data not shown). The vectors PCMV-CYP11B2 or PCMV-CYP11B1/B2 used in the transfection experiments with HEK-293 cells have the same viral promoter. For that reason, the expression levels of chimeric or wild type aldosterone synthase should be affected by the same effectors. Data in Figure 3 are expressed as the mean +/− S.E.M. of four independent trials.

### Molecular modelling of CYP11B1 and ASCE proteins

**Figure 4:**
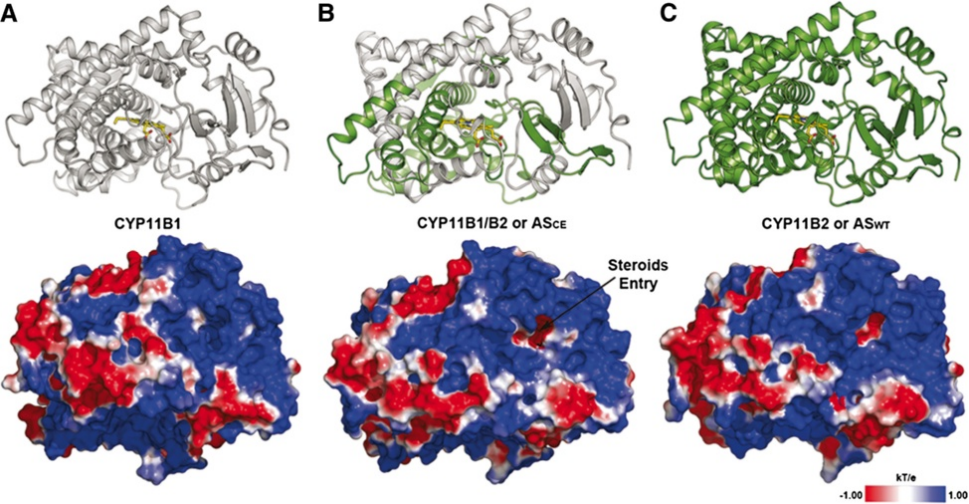
**Molecular modelling of CYP11B1 and ASCE proteins.** Schematic representation of the secondary structures and electrostatic potential profiles of CYP11B1 (11BH, Panel **A**), CYP11B1/B2 (ASCE, Panel **B**) and CYP11B2 (ASWT, Panel **C**). The ASCE secondary structure is colored according to crossover occurring at helix G, where grey represents the CYP11B1 portion and green represents the CYP11B2 portion of the chimeric enzyme. The solvent accessible surface colored according to the calculated electrostatic potential shows that the steroid binding site in ASCE is more electronegative and more extended than that of the ASWT (553.62 v/s 471.87 Å^3^).

We developed 3D models of both proteins by comparative modelling using the human CYP11B2 (ASWT) crystal structure. In the final alignment used to model the proteins, the percentage of sequence identity was 93.6% and 97.7% for the modelled region, and 96.4% and 98.9% homologies were observed between the template structure and CYP11B1 and ASCE, respectively. For ASCE, the grey bar indicates the corresponding CYP11B1 portion, and the green bar represents the CYP11B2 limits for ASCE (See Additional file 3: Figure S3). A Ramachandran plot analysis indicated that the modelled proteins have more than 95% of the residues in the allowed region. The PROSA Z-scores and Verify3D profile scores also support the quality of the obtained models for further studies (Table 1). The obtained models exhibit a root-mean square deviation (RMSD) of alpha carbons of less than 0.4 Å when superimposed on the template structure. Figure 4 depicts the secondary structure of the modelled proteins compared to ASWT. The ASCE model (Figure 4B) is composed of the first 38% of the 11BH (residues 34 to 191), which contains the substrate (steroid) entry region of CYP11B1, and the last 62% of the ASWT (residues 192–503). All models have the common folding pattern of CYP450 enzymes, and according to the identified crossover, the shift will occur at the end of helix G. Electrostatic potential surface profiles of the proteins indicate that the surface potential of ASCE is more similar to that of ASWT, with the steroid binding site access channel in ASCE being more electronegative and more extended than that of ASWT, as determined by the size of the associated cavities, which are 471.87 and 553.62 Å^3^ for ASCE and ASWT, respectively (Table 2).

**Table 1:** Protein modelling validation summary

**Protein**	**DOPE score**	**RMSD (Å)**^**a**^	**Ramachandran statistics (% of residues)**			**Verify 3D**			**PROSA Z-Score**	**Cavity volume (Å**^**3**^**)**
			**Favoured**	**Allowed**	**Outlier**	**Verify score**	**Max verify score**	**Min verify score**		
*CYP11B2*	NA	0.00	99.6	0.2	0.2	204.69	214.638	96.58	−9.50	471.87
*CYP11B1*	−58932.24	0.24	96.6	0.2	0.2	196.41	214.178	96.38	−9.97	515.12
*CYP11B1/B2*	−58732.37	0.32	97.2	1.5	1.3	195.07	214.178	96.38	−10.13	553.62

^*a*^*: RMSD (root-mean square deviation) from Template CYP11B2 (PDB id 4DVQ, Chain A).*

**Table 2:** Summarised docking results

**Ligand**	**CYP11B2**		**CYP11B1/B2**	
	**ChemGauss4 Score**	**ΔGbind (kcal/mol)**	**ChemGauss4 Score**	**ΔGbind (kcal/mol)**
*DOC*	−16.58	−7.09	−18.70	−7.32
*Ketoconazole*	−17.66	−8.92	−19.65	−9.63
*Progesterone*	−15.78	−6.52	−17.98	−6.90
*Estradiol*	−14.06	−6.26	−16.12	−5.44

ΔGbind: Calculated as Ludi2 Score /-73.33 (kcal/mol).

### Type of inhibition and docking of steroids to CYP11B1, ASCE and ASWT proteins

**Figure 5:**
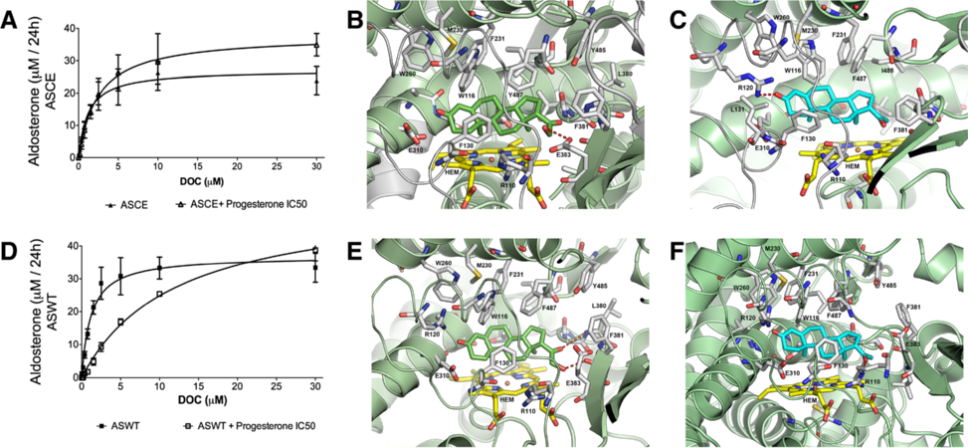
**Type of inhibition and docking of steroids to ASCE and ASWT proteins.** The new calculated kinetic enzyme parameters obtained in the presence of the corresponding IC50 concentration of progesterone for each enzyme were Km = 2.451 μM and Vmax = 37.91 μM/24 h for ASCE, and Km = 10.78 μM and Vmax = 51.61 μM/24 h for ASWT. Progesterone inhibits the aldosterone synthase wild type in a competitive fashion. **(A** and **D)**. Docking experiments show that DOC binds in a similar mode to ASCE compared to ASWT **(B** and **E)**. Progesterone also has a similar binding mode to ASCE and ASWT, but penetrates deeper into the ASCE pocket **(C** and **F)**.

To gain insight into the inhibition profile of progesterone, we performed kinetic experiments in the presence of the corresponding IC50 concentrations of progesterone for each enzyme (Figures 5A and 5D). In progesterone presence, ASCE Km was very variable and lightly increase, Vmax was no statistically different (p = 0.0667). ASWT Km significantly increase (p = 0.0095) but Vmax was no statistically different (p = 0.1048). The new calculated kinetic enzyme parameters obtained in presence of progesterone were Km = 2.451 μM and Vmax = 37.91 μM/24 h for ASCE, and Km = 10.78 μM and Vmax = 51.61 μM/24 h for ASWT. Progesterone inhibits the aldosterone synthase wilde type in a competitive fashion.

To explore the putative binding mode of the steroid compounds in CYP11B1, ASCE and ASWT, we performed docking simulations within the binding site of all proteins. Figure 5B and C show the most favourable predicted binding mode obtained for DOC and progesterone within the ASCE, where progesterone binds with its 3-carboxy group facing the inner part of the binding pocket that is composed of the side chains of Arg120 and Glu310. The composition of this binding pocket is similar to that of DOC in ASCE and ASWT (Figure 5B and E, respectively), but progesterone penetrates deeper into the pocket with stabilisation via a hydrogen bond interaction with Arg120. The methyl groups at positions 18 and 19 face the HEME group and are surrounded by the side chains of Ala313 and Thr318. The beta side of the steroid scaffold faces the top of the aromatic cluster pocket composed by Trp116, Trp260, Phe231 and Phe487. Figure 5F represents the most favourable predicted binding mode obtained for progesterone with ASWT.

DOC and progesterone have a similar binding mode in CYP11B1 as that predicted for ASCE (See Additional file 4: Figure S4A and Figure S4B) and as the experimentally determined binding mode of DOC to ASWT (Figure 5B). Estradiol binds with its aromatic portion positioned inside the pocket and interacts with the pocket via its aromatic portion with the side chains of Phe130, Trp116 and Trp260 but fails to establish any H-bond interactions with the binding site (See Additional file 4: Figure S4C). Finally, ketoconazole binds with its imidazole moiety making direct contact with the iron atom in the HEME group, with its dichlorophenyl group making aromatic interactions with Phe130 and Trp116, and with its terminal amide in making H-bond interactions with Asn404 (See Additional file 4: Figure S4D). The binding energies displayed in Table 2 suggest that sex steroids can bind to both proteins, ASCE and ASWT, but with lower affinity than ketoconazole or DOC to ASWT, according to the ChemGauss4 scoring and Ludi2 scoring function-derived binding energies.

## Discussion

In this work, we demonstrated for the first time that progesterone inhibits ASCE and ASWT but that estradiol had no effect in our system. To probe this, we successfully generated an *in vitro* system expressing ASCE and ASWT in transfected HEK-293 cells. Moreover, in our *in vitro* model, we assumed that both enzymes were expressed in similar amounts because the percent of cells transfected was similar when using different vectors. *In vitro* aldosterone production following increasing DOC concentration was reproducible and comparable in independent trials for both enzymes, with similar Vmax and Km. Progesterone inhibited the ASCE with lower potency than but similar efficacy as ASWT. This is a novel result because previous reports have demonstrated that progesterone reduces the blood pressure via an antagonising effect on mineralocorticoid receptors and peripheral vasorelaxation [38-40].

We found that progesterone inhibited both ASCE and ASWT, which is in agreement with studies from the Marquet group that showed that progesterone derivatives inhibit ASWT activity [41,42]. However, these findings are in contrast to published studies on animal models, where progesterone increased aldosterone production by ASWT [17] and increased mRNA CYP11B2 production [17]. In our system, estradiol showed no effect on both ASCE and ASWT activities in contrast to the results reported by Kau, where an increase in the production of aldosterone by estradiol replacement in ovariectomised rat was shown [43].

Modelling studies support the competitive inhibitory effect of progesterone observed *in vitro*, which shows that DOC, the natural substrate, and progesterone have a similar binding mode within the active site of both enzymes. DOC and progesterone interact with the same amino acids of the active site on ASCE and ASWT, which is in agreement with the aldosterone production obtained by both enzymes *in vitro*. However, the modelling analysis showed that the steroid entrance loop in ASCE corresponds to CYP11B1, which is different from ASWT. Interestingly, a feature of ASCE is that it contains all of the substitutions reported to convert CYP11B1 into an aldosterone synthase enzyme, such as S288G, V320A and N335D [12,44]. The docking simulations also support that non-aromatic sex steroids, such as progesterone, possessing a structure similar to the endogenous substrate, bind to the steroid binding pocket with higher affinity than aromatic sex steroids, such as estradiol. Conformational flexibility is an important feature for substrate specificity and for the positioning of substrates within the binding pocket for further enzymatic processing; therefore, the planar aromatic ring of estradiol will reduce its conformational flexibility and binding to the steroid cavity in ASCE and ASWT.

According to our *in vitro* and *in silico* results, we would expect that the plasma aldosterone concentration should decrease as progesterone levels increase alongside gestation. On the contrary, it is generally accepted that, in pregnancy, plasma aldosterone and progesterone concentrations increase. How does one solve this discrepancy? Some authors have speculated that the mineralocorticoid receptor (MR), under normal circumstances, may be protected from activation by DOC via a pre-receptor protective mechanism, although the nature of which is unclear. We believe that during gestation there is a close relationship between aldosterone and progesterone levels, which must be carefully regulated alongside pregnancy to prevent the onset of hypertension and to have a successful delivery and a healthy newborn. We postulated that progesterone may have a multifactorial role on aldosterone production: on one hand, it would favour aldosterone synthesis by acting as a substrate for adrenal 21-hydroxylase [16] or by increasing the expression of CYP11B2 mRNA levels [17], and on the other hand, it would inhibit aldosterone synthase activity, thereby protecting the mother not only from the systemic effects of aldosterone production but also from the devastating local effects of aldosterone production.

## Conclusions

In summary, we have determined the kinetic parameters for ASCE and ASWT in our *in vitro* model, and we have demonstrated that progesterone but not estradiol inhibited the aldosterone synthase activity of ASCE and ASWT *in vitro*_._ This finding suggests a novel pre-receptor mechanism of control for aldosterone levels by progesterone, differing from the previously described mechanisms, such as binding to the mineralocorticoid receptor. This mechanism may operate as a buffering system in pregnant women where high levels of progesterone are produced by the placenta, suggesting that this mechanism could be important in preventing the deleterious effects of aldosterone on vasculature.

## Abbreviations

ASCE: Chimeric aldosterone synthase enzyme; ASWT: Wild type aldosterone synthase enzyme; CYP11B1: 11β-hydroxylase gene, 11BH, 11β-hydroxylase protein; CYP11B1/B2: Chimeric gene; CYP11B2: Aldosterone synthase gene; DOC: 11OH-Deoxicorticosterone; FH-I: Familial hyperaldosteronism type I; HEK-293: Human embryonic kidney cells; HPLC-MS/MS: high performance liquid chromatography coupled with tandem mass spectrometry; PCMV: Cytomegalovirus promoter.

## Competing interests

The authors declare that they have no competing interests.

## Authors’ contributions

AV, CFL, CAF, FA, CC, SS, CAC and CEF made substantial contributions to the conception and design of the experiments. AV, CFL, CAF and FA made substantial contributions to the acquisition of data. AV and CAF performed the chimeric design, plasmid amplification, transfection and cellular manipulation. FA, SS, CV and AT participated in the measuring of aldosterone content by HPLC-MS/MS. KM and TO participated in the Western blot result. CFL performed the molecular modelling and analysed the data. AV, CFL, CC, GO, CAC and CEF made substantial contributions to the analysis and interpretation of data. AV, CFL, CC and CEF were involved in drafting the manuscript, and AV, CFL, CC, CEF, GO and CAC were involved in critically revising the manuscript for important intellectual content. All authors read and approved the final manuscript.

## Supplementary Material

Additional file 1: Figure S1 Quantitive RT-PCR of No transfected (NT) or CYP11B2 or CYP11B1/B2 transfected HEK-293 cell and incubated with different progesterone concentration (0.625 to 5 μM). There were no differences in mRNA expression by progesterone respect to each control condition (without progesterone). Click here for file

Additional file 2: Figure S2 CYP11B2 or CYP11B1/B2 transfected HEK-293 cell were incubated with different combination of estradiol/progesterone concentration. Different dose response for aldosterone production by ASCE (A) and ASWT (B) (μM/24 h). Estradiol had no additional inhibitory effect on wild type or chimeric aldosterone synthase activity when was co-incubated with progesterone. Click here for file

Additional file 3: Figure S3 Sequence alignment used to model proteins CYP11B1/B2 (ASCE) and CYP11B1 using human CYP11B2 (ASWT) as template. The percentage of sequence identity was 93.6% and 97.7% for the modelled region, and 96.4% and 98.9% homologies were observed between the template structure and CYP11B1 and ASCE, respectively. For ASCE, the grey bar indicates the corresponding CYP11B1 portion, and the green bar represents the CYP11B2 limits for ASCE. Click here for file

Additional file 4: Figure S4 The 11OH-deoxycorticosterone (DOC) and progesterone predicted binding mode to CYP11B1 (A and B, respectively). Estradiol binding mode to ASCE (C) and ketoconazole binding to ASWT binding pocket (D). Click here for file
