# Supplementary figures and images for: Different effects of progesterone and estradiol on chimeric and wild type aldosterone synthase in vitro
Source: Reprod Biol Endocrinol. 2013 Aug 13;11:76. doi: 10.1186/1477-7827-11-76 (PMC3848474; doi:10.1186/1477-7827-11-76)

CYP11B2/18S  $\Delta\Delta Ct$

120000  
90000  
60000  
30000  
3  
2  
1  
0

Progesterone ( $\mu M$ )

0 0,6 1,2 2,5 5

NT

0 0,6 1,2 2,5 5

CYP11B2

0 0,6 1,2 2,5 5

CYP11B1/B2

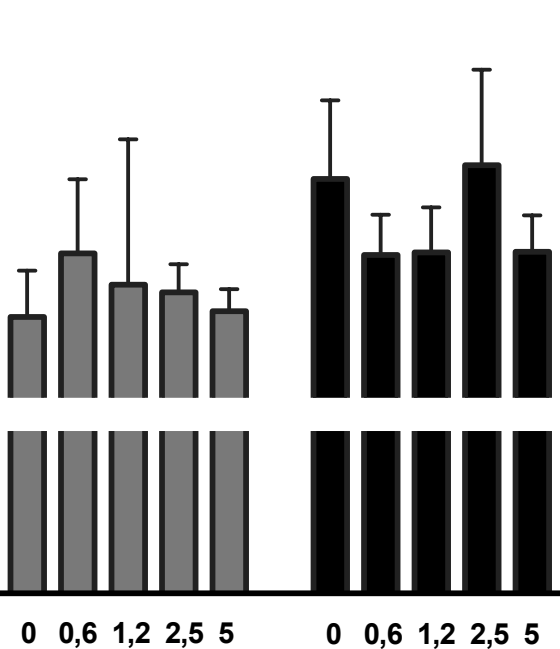

Supplement: Additional file 1: Figure S1 — Quantitive RT-PCR of No transfected (NT) or CYP11B2 or CYP11B1/B2 transfected HEK-293 cell and incubated with different progesterone concentration (0.625 to 5 μM). There were no differences in mRNA expression by progesterone respect to each control condition (without progesterone). [file 1477-7827-11-76-S1.pdf]

**A**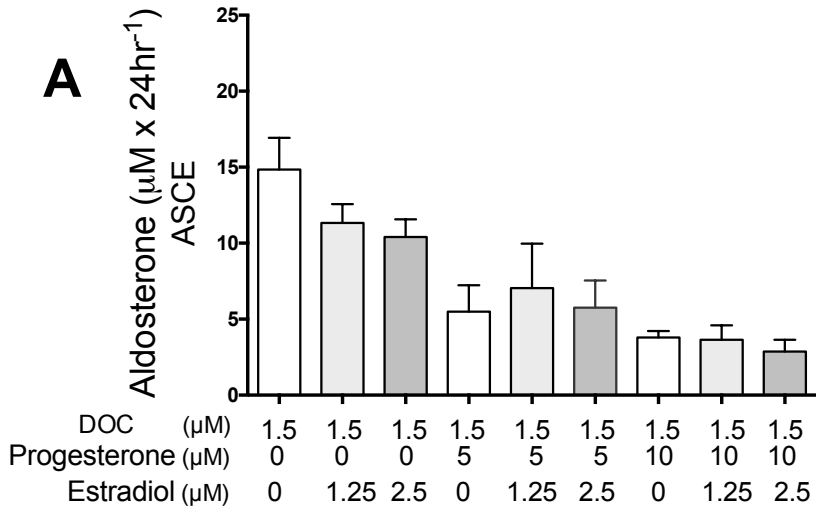**B**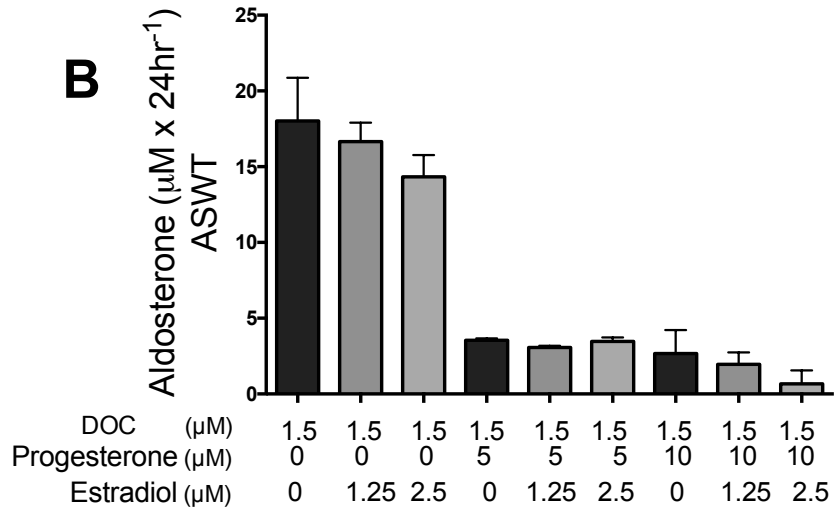

Supplement: Additional file 2: Figure S2 — CYP11B2 or CYP11B1/B2 transfected HEK-293 cell were incubated with different combination of estradiol/progesterone concentration. Different dose response for aldosterone production by ASCE (A) and ASWT (B) (μM/24 h). Estradiol had no additional inhibitory effect on wild type or chimeric aldosterone synthase activity when was co-incubated with progesterone. [file 1477-7827-11-76-S2.pdf]

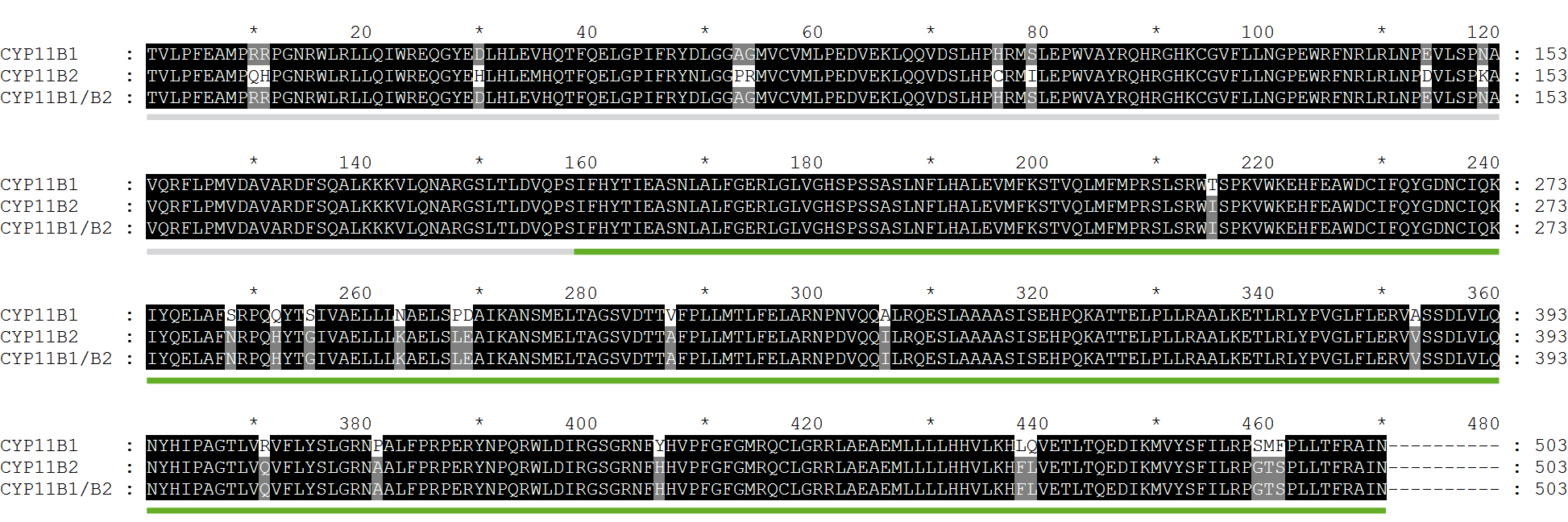

Supplement: Additional file 3: Figure S3 — Sequence alignment used to model proteins CYP11B1/B2 (ASCE) and CYP11B1 using human CYP11B2 (ASWT) as template. The percentage of sequence identity was 93.6% and 97.7% for the modelled region, and 96.4% and 98.9% homologies were observed between the template structure and CYP11B1 and ASCE, respectively. For ASCE, the grey bar indicates the corresponding CYP11B1 portion, and the green bar represents the CYP11B2 limits for ASCE. [file 1477-7827-11-76-S3.jpeg]

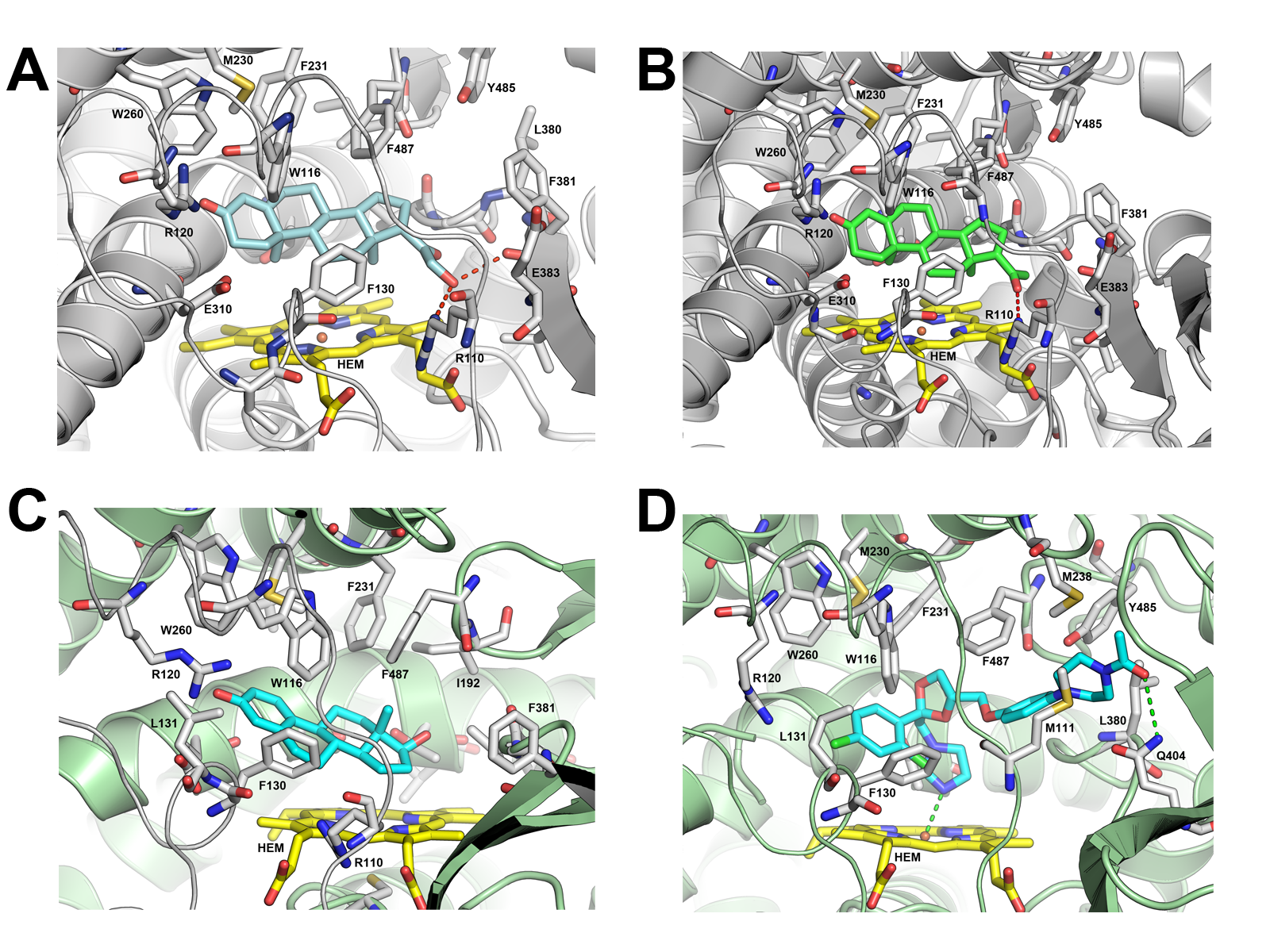

Supplement: Additional file 4: Figure S4 — The 11OH-deoxycorticosterone (DOC) and progesterone predicted binding mode to CYP11B1 (A and B, respectively). Estradiol binding mode to ASCE (C) and ketoconazole binding to ASWT binding pocket (D). [file 1477-7827-11-76-S4.tiff]
